# The role of thromboelastometry in the assessment and treatment of coagulopathy in liver transplant patients

**DOI:** 10.1590/S1679-45082017MD3903

**Published:** 2017

**Authors:** Raffael Pereira Cezar Zamper, Thiago Chaves Amorim, Luiz Guilherme Villares da Costa, Flávio Takaoka, Ary Serpa

**Affiliations:** 1 Hospital Israelita Albert Einstein, São Paulo, SP, Brazil.

**Keywords:** Transplantation, Liver transplantation, Blood coagulation, Thrombelastography, Hemorrhage, Blood transfusion

## Abstract

Perioperative monitoring of coagulation is vital to assess bleeding risks, diagnose deficiencies associated with hemorrhage, and guide hemostatic therapy in major surgical procedures, such as liver transplantation. Routine static tests demand long turnaround time and do not assess platelet function; they are determined on plasma at a standard temperature of 37°C; hence these tests are ill-suited for intraoperative use. In contrast, methods which evaluate the viscoelastic properties of whole blood, such as thromboelastogram and rotational thromboelastometry, provide rapid qualitative coagulation assessment and appropriate guidance for transfusion therapy. These are promising tools for the assessment and treatment of hyper- and hypocoagulable states associated with bleeding in liver transplantation. When combined with traditional tests and objective assessment of the surgical field, this information provides ideal guidance for transfusion strategies, with potential improvement of patient outcomes.

## RESUMO

A monitorização perioperatória da coagulação é fundamental para estimar o risco de sangramento, diagnosticar deficiências causadoras de hemorragia e guiar terapias hemostáticas durante procedimentos cirúrgicos de grande porte, como o transplante hepático. Os testes estáticos, comumente usados na prática clínica, são insatisfatórios no intraoperatório, pois demandam tempo e não avaliam a função plaquetária; são determinados no plasma e realizados em temperatura padrão de 37°C. Os métodos que avaliam as propriedades viscoelásticas do sangue total, como o tromboelastograma e a tromboelastometria rotacional, podem suprir as deficiências dos testes estáticos tradicionais, uma vez que permitem avaliar a coagulação de forma rápida e qualitativa, guiando a terapia transfusional de forma adequada. A tromboelastometria rotacional mostrou-se promissora na avaliação e no tratamento de estados de hipercoagulação e hipocoagulação, associados a sangramento no transplante hepático. Estas informações, combinadas com os testes tradicionais e uma avaliação objetiva do campo cirúrgico, promovem um cenário ótimo para guiar as estratégias transfusionais e potencialmente melhorar o desfecho destes pacientes.

## INTRODUCTION

Perioperative monitoring of blood coagulation is vital to assess bleeding risks, diagnose deficiencies associated with hemorrhage and guide hemostatic therapy during major surgical procedures, such as liver transplantation. ^^1^^ Routine tests performed under static conditions (prothrombin time, international normalized ratio, activated partial thromboplastin time, fibrinogen levels and platelet count) have long turnaround time, do not assess platelet function, are conducted on plasma rather than whole blood, at 37°C, which often does not reflect the actual temperature of the patient; ^^2^^ therefore such tests are ill-suited for dynamic intraoperative work settings. ^^2^^ Methods that measure the viscoelastic properties of whole blood, such as thromboelastogram (TEG^®^) and rotational thromboelastometry (ROTEM^®^), can be used to guide transfusion therapy in cirrhotic patients; these methods provide fast qualitative assessment of coagulation and can overcome the limitations of traditional static tests. ^^3^^

Coagulation protein synthesis is often impaired in patients with liver disease. These changes can be counteracted by mechanisms leading to a new hemostatic equilibrium. ^^4^^ The major mechanisms include (1) dysfunction and impaired production of pro- and anticoagulation factors leading to bleeding, thrombosis or relative hemostatic equilibrium in patients with end-stage liver disease; ^^5^^ and (2) decrease in circulating platelets due to splenic sequestration, faster platelet turnover, shortened platelet half-life or decreased platelet production (low thrombopoietin levels), which is counteracted by increased secretion of von Willebrand factor, a platelet adhesion mediator.

Patients suffering from cirrhosis are deficient in procoagulant factors II, V, VII, IX, X and XI. These deficiencies affect routine laboratory based coagulation tests, particularly prothrombin time, international normalized ratio and activated partial thromboplastin time. However, in spite of decreased procoagulant factor levels, cirrhotic patients may still have normal thrombin generating capacity due to decreased production of protein C (a potent anticoagulant) and increased levels of endothelium-derived factor (factor VIII). ^^6^^

## VISCOELASTIC METHODS OF COAGULATION ASSESSMENT

**Figure 1:**
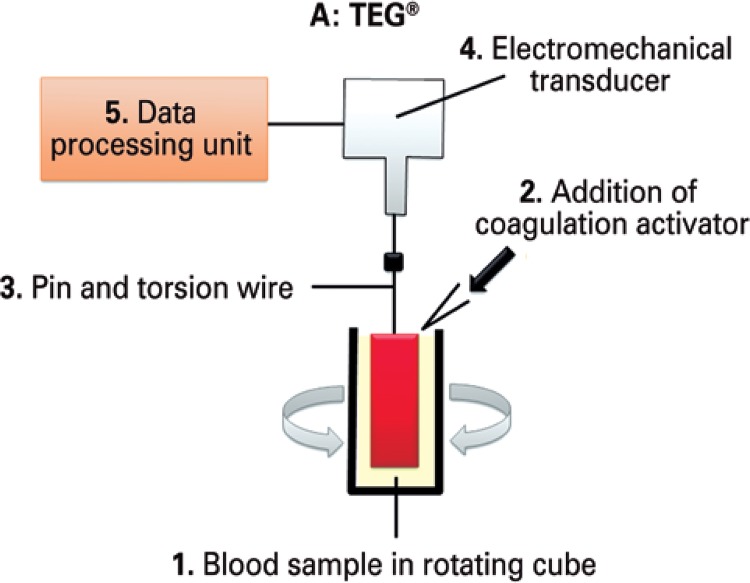
Working principle behind TEG®

Thromboelastography was originally described in 1948 as a method for global assessment of hemostatic function using a sample of blood. Different from conventional tests, TEG^®^ is a whole blood based assay run at the temperature of the patient, and therefore allows the assessment of platelet function and platelet-erythrocyte interactions. Thromboelastogram (Haemoscope/Haemonetics^®^, Niles, Ill) enables a comprehensive dynamic assessment of the coagulation process; in this test, a blood sample is loaded into a stationary cup that rotates through 4^o^45’ in 10-second cycles. Movement is then monitored via a pin suspended into the blood sample following addition of a coagulation activator (Figure 1).

TEG^®^: thromboelastogram.

**Figure 2:**
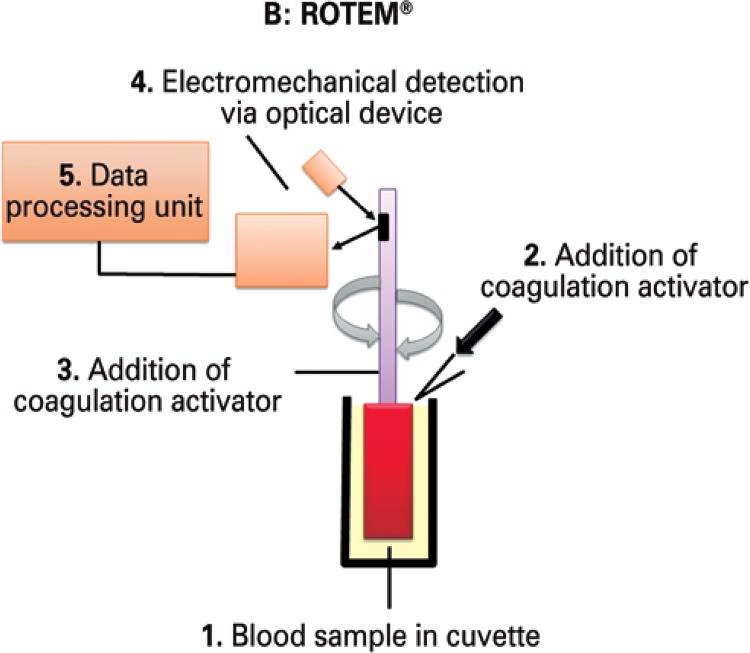
Working principle behind ROTEM®

**Figure 3:**
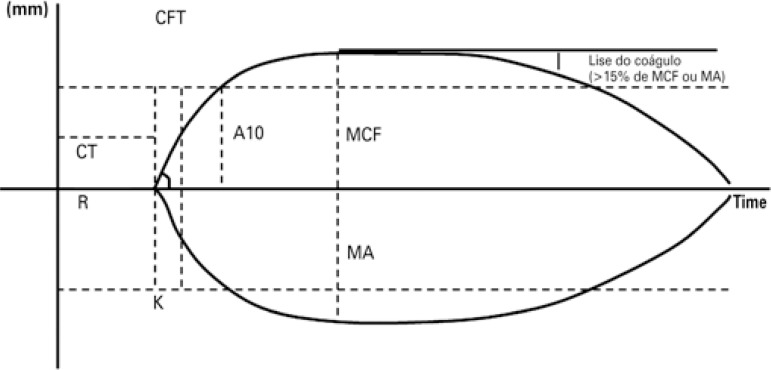
Whole blood viscoelastic tests can be detected using ROTEM® (optical method, upper line) or TEG® (electromechanical method; bottom line). Plasmatic coagulation can be detected by means of coagulation or reaction time. Initial clot formation is indicated by clot formation time (k time) and a angle. Maximum viscoelasticity is defined as maximum clot firmness or maximum amplitude. Systemic fibrinolysis is indicated by clot lysis (>15% of maximum amplitude or maximum clot firmness) within 1 hour. A10: amplitude 10 minutes post clotting time

CT: clotting time; R: reaction time; CFT: clot formation time; K: K time; MCF: maximum clot firmness; MA: maximum amplitude.

The ROTEM^®^ device is a modification of the TEG^®^ technology based on the same working principles: the signal generated by the suspended pin is transmitted via optical detection system instead of a torsion wire, and movement is generated by the pin, not the cup (Figure 2). Both TEG^®^ and ROTEM^®^ measure and provide graphic representation of viscoelastic changes across all stages of clot formation, persistence and resolution (Figure 3).

ROTEM^®^: rotational thromboelastometry.

Reagents specific to ROTEM^®^ enable assessing different aspects of the coagulation process and thus provide guidance for correction of potential disorders, ^^7^^ a major advantage over TEG^®^. The following variations of the assays are used to assist decision making concerning transfusion in clinical practice: (1) EXTEM (tissue factor activation; rapid assessment of clot formation and fibrinolysis via the extrinsic coagulation pathway); (2) INTEM (contact activation; assessment of clot formation and fibrin polymerization via the intrinsic coagulation pathway); (3) FIBTEM (tissue factor activation combined with platelet inhibitor cytochalasin D; qualitative assessment of fibrinogen levels); (4) APTEM (tissue factor activation combined with aprotinin; assessment of the fibrinolytic pathway; rapid detection when combined with EXTEM); and (5) HEPTEM (contact activation combined with heparinase; detection of heparin or heparinoids in the sample).

Assuming optimal conditions for hemostasis (temperature, blood pH and calcium serum levels within normal ranges) and clinical diagnosis of coagulopathy based on objective assessment of the surgical field, viscoelastic methods can be used to guide clotting factor or platelet replacement.

## VISCOELASTIC METHODS IN LIVER TRANSPLANTATION

Increased rates of infection and hepatic artery thrombosis after liver transplantation have been associated with red blood cell transfusion. All blood products (cryoprecipitate, fresh frozen plasma and platelets) were shown to have negative impacts on 1 and 5-year graft survival. Also, transfusion of cryoprecipitate, fresh frozen plasma and/or platelets is associated with transfusion related acute lung injury. ^^8^^

In contrast with viscoelastic tests, conventional coagulation tests do not reflect the actual hemostatic status of cirrhotic patients. The limitation in ROTEM^®^ is that the test is run with blood outside the endothelium under no-flow conditions, which precludes abnormal results to be interpreted as indicative of coagulopathy in patients without evident bleeding. ^^9^^

Hence, ROTEM^®^ is used as a guide for blood product replacement in patients with signs of coagulopathy and bleeding of non-surgical origin, provided temperature, blood pH and calcium serum levels are within normalcy values. ^^2^^ Rotational thromboelastometry is thought to be a promising tool for assessment and treatment of hyper- and hypocoagulable states associated with bleeding during major surgical procedures, such as liver transplantation. ^^10^^

## CONCLUSION

Thromboelastogram and rotational thromboelastometry deliver valuable real time information for perioperative coagulopathy management across the different phases of liver transplantation. When combined with traditional tests and objective assessment of the surgical field, these tools provide ideal guidance for transfusion strategies and have the potential to improve patient outcomes. Further studies are warranted to determine which parameters to target and which transfusion triggers to use for improved perioperative management of patients undergoing liver transplantation.
